# Apolipoprotein E Interferes with IAPP Aggregation and Protects Pericytes from IAPP-Induced Toxicity

**DOI:** 10.3390/biom10010134

**Published:** 2020-01-14

**Authors:** Anna L. Gharibyan, Tohidul Islam, Nina Pettersson, Solmaz A. Golchin, Johanna Lundgren, Gabriella Johansson, Mélany Genot, Nina Schultz, Malin Wennström, Anders Olofsson

**Affiliations:** 1 Department of Medical Biochemistry and Biophysics, Umeå University, SE-901 87 Umeå, Sweden; tohidul.islam@umu.se (T.I.); ninalinneap@gmail.com (N.P.); s.a.golchin@gmail.com (S.A.G.); johanna_jem3@hotmail.com (J.L.); gjoh0007@student.umu.se (G.J.); melany.genot@gmail.com (M.G.); 2 Clinical Memory Research Unit, Department of Clinical Sciences Malmö, Lund University, 21428 Malmö, Sweden; nina.schultz@med.lu.se (N.S.); malin.wennstrom@med.lu.se (M.W.)

**Keywords:** apolipoprotein E, IAPP amyloid, Thioflavin T, pericytes, cytotoxicity

## Abstract

Apolipoprotein E (ApoE) has become a primary focus of research after the discovery of its strong linkage to Alzheimer’s disease (AD), where the ApoE4 variant is the highest genetic risk factor for this disease. ApoE is commonly found in amyloid deposits of different origins, and its interaction with amyloid-β peptide (Aβ), the hallmark of AD, is well known. However, studies on the interaction of ApoEs with other amyloid-forming proteins are limited. Islet amyloid polypeptide (IAPP) is an amyloid-forming peptide linked to the development of type-2 diabetes and has also been shown to be involved in AD pathology and vascular dementia. Here we studied the impact of ApoE on IAPP aggregation and IAPP-induced toxicity on blood vessel pericytes. Using both in vitro and cell-based assays, we show that ApoE efficiently inhibits the amyloid formation of IAPP at highly substoichiometric ratios and that it interferes with both nucleation and elongation. We also show that ApoE protects the pericytes against IAPP-induced toxicity, however, the ApoE4 variant displays the weakest protective potential. Taken together, our results suggest that ApoE has a generic amyloid-interfering property and can be protective against amyloid-induced cytotoxicity, but there is a loss of function for the ApoE4 variant.

## 1. Introduction

Apolipoprotein E (ApoE) is a lipid transport protein that exists in the three isoforms ApoE2, ApoE3, and ApoE4 that are encoded by the three alleles ε2, ε3, and ε4, respectively [1]. These have attained a lot of focus since the discovery of their strong genetic linkage to Alzheimer’s disease (AD), with the ApoE4 variant being the highest genetic risk factor for the development of the disease [1,2,3]. The amino acid differences between the three isoforms of ApoE are limited to positions 112 and 158, where ApoE2 has Cys residues at both positions; ApoE3 has a Cys at position 112 and an Arg at position 158, and ApoE4 has Arg residues at both of these positions [1,4,5]. These seemingly small differences in the sequence have a great impact on ApoE functionality [1,6]. The information about the structural and conformational differences between the ApoE variants is controversial. Some studies show that ApoE4 differs from ApoE2 and ApoE3 by obtaining a more open conformation [7,8], which in turn affects its lipid- and receptor binding ability. Moreover, differences in stability and conformation between the ApoEs have been suggested [9,10], indicating that ApoE4 is less stable and can aggregate into amyloid-like fibrils [10]. However, a recent study by Raulin et al. shows that there are no significant differences between the recombinant ApoE variants at the structural or conformational level, although ApoE4 has a higher propensity to form non-amyloid aggregates [6].

The allele frequency of the isoforms in the global population is 5% to 10% for ApoE2, 65% to 70% for ApoE3, and 15% to 20% for the ApoE4 variant [11], which give rise to 6 biallelic genotypes (ε3/ε3 > ε3/ε4 > ε2/ε3 > ε4/ε4 > ε2/ε4 > ε2/ε2) [12]. Apoε4 homozygotes have about 10-fold higher risk for developing AD and about 40% to 65% of all AD cases are carriers of at least one copy of Apoε4 [13]. Consequently, the relative prevalence of co-localization of ApoE together with Aβ-amyloid plaques is ApoE4 > ApoE3 > ApoE2 [14,15]. These findings led to numerous studies seeking to elucidate the role of ApoE on Aβ amyloid formation and related AD pathology [3,15,16,17,18,19,20,21,22,23,24,25]. Together with a handful of other endogenous proteins with amyloid interfering properties, such as transthyretin [26,27], proteins with a BRICHOS domain [28] and clusterin [29], it has been shown that ApoE can directly interfere with the Aβ aggregation process by inhibiting or slowing down fibril formation [3,16,19,20,21,24,30]. However, the impact of such interference on disease progression is still not fully understood and the role in the process of amyloid formation remains controversial. Studies on AD mouse models have shown that all ApoE-carrying AD mice develop amyloid plaques with the highest load observed in ApoE4 animals, while the complete knockout of the *ApoE* gene, in essence, eliminates plaque formation and any signs of disease [14,31]. A recent finding also showed efficient clearance of Aβ plaques by the administration of ApoE-specific antibodies [32]. These findings suggest that ApoE acts as a pathological chaperone, although to different extents depending on the ApoE variant. In contrast, several works have suggested that ApoE plays an important role in the degradation and clearance of Aβ amyloid [15,17,33,34]. Recently, it has been shown that ApoE protects human pericytes against Aβ-induced cytotoxicity [18] and maintains a receptor-mediated in-pericyte clearance of Aβ aggregates [23], and both of these studies showed that ApoE4 had significantly weaker effects than the other two variants.

The clinical importance of ApoE and its ability to modulate both amyloid formation and disease progression recently gained further support in a case report, showing a strong protective effect of ApoE3 Christchurch variant on the background of an aggressive familial presenilin mutation, expected to result in early-onset Alzheimer’s disease [35].

Apart from structural and functional differences, it has been shown that serum concentrations of ApoE isoforms are substantially different and that ApoE4 expression is the lowest (frequently only around 20% compared to the other *ApoE* alleles) [36,37]. Lower ApoE expression has also been observed in pericytes with the ε4 genotype, and this is accompanied by higher vulnerability to Aβ toxicity compared to non-ε4 pericytes [18]. These findings imply that ApoE, in general, has a protective role against amyloid formation and toxicity, with loss of function for ApoE4. In addition, it has been shown that a low level of ApoE is a general risk factor for AD irrespective of isoform [38,39].

Islet amyloid polypeptide (IAPP) (also known as amylin) is another highly amyloidogenic peptide and deposition in pancreatic islets is tightly linked to type-2 diabetes [40,41,42,43]. In addition, an increasing number of experimental and clinical data indicate that IAPP aggregation can occur also in the brain, vascular system, heart, and kidneys [44,45,46]. IAPP deposition in the brain of type-2 diabetes patients with dementia and patients with AD [44,47] led to the hypothesis that IAPP along with Aβ is involved in AD pathology [44]. Animal studies showed also that overexpression of human IAPP in rats leads to neurological deficits and neuroinflammation [48].

Interestingly, similar to findings in Aβ plaques, ApoE was found to be co-deposited with IAPP amyloid [49,50,51]. However, the experimental data on the interaction between IAPP and ApoE are very scarce [52] in spite of the apparent importance of ApoE in IAPP-related pathologies [11,49,50,51] and the crosslink between IAPP pathologies and AD as well as other types of dementia [44,47].

Similar to other amyloid peptides and proteins, IAPP aggregates induce cell and tissue degeneration [53]. The exact mechanism by which IAPP induces cytotoxicity remains unknown, but some experimental data suggest that IAPP aggregates on the cell surface leading to membrane disruption and leakage [54], induces oxidative stress [55], and impairs the autophagy/lysosomal degradation system in the cells [56,57], all of which play key roles in cytotoxicity.

In a recent study, it was shown that IAPP forms intracellular inclusions in brain microvessel pericytes, leading to nuclear changes and loss of neuron-glial antigen 2, which is an important protein for pericyte proliferation, migration, and survival [53]. Pericytes are key components of the neurovascular system in the brain, where they maintain homeostatic and hemostatic functions by regulating capillary blood flow, the permeability of the blood-brain barrier, and clearance of cellular debris [58]. Given that pericyte deficiency or dysfunction is associated with a number of central nervous system disorders, including diabetic retinopathy [59] and neurodegenerative disorders such as AD [60], these findings [53], together with the studies on Aβ-ApoE effects on pericytes [18,23], make pericytes an interesting model for studying the influence of ApoE on IAPP-induced toxicity.

In the current work, we have investigated the interfering properties of ApoE variants with IAPP amyloid formation in vitro and assessed the role of ApoE on IAPP-induced toxicity on cultured human blood vessel pericytes. We show that ApoE interferes with both nucleation and elongation during IAPP amyloid formation. The efficacy of the different isoforms in the in vitro setups was essentially the same. However, despite the lack of a significant difference between the three ApoE variants in terms of interfering with IAPP aggregation, they protected the pericytes against IAPP-induced toxicity in an isotype-dependent manner with the weakest effect observed for ApoE4. Given the fact that ApoE4 is also in general found at significantly lower concentrations in human serum, [37,39] the effect is pronounced. Taken together, we propose a mechanistic route for IAPP interference by ApoE and an allelic difference regarding its ability to protect against IAPP-induced cytotoxicity in pericytes.

## 2. Materials and Methods

### 2.1. Preparation of ApoE Isoforms and IAPP

ApoE isoforms and synthetic human IAPP were obtained from AlexoTech AB (Umeå, Sweden). All proteins were at high quality as verified by LC-MS analysis (Figure S6). IAPP contained a disulfide bridge between the positions 2 and 7 and was amidated at C-terminal. Prior to use, the lyophilized IAPP was dissolved in 5% acetic acid and 150 mM NaCl and ApoE variants were dissolved in 20mM NaOH to eliminate potential aggregated, then subjected to size-exclusion chromatography on a Superdex 75 Increase 10/300 GL column, with elution in PBS containing 2 mM EDTA and 0.02% NaN_3_. Fractions containing monomeric IAPP were collected for further analysis.

### 2.2. Thioflavin-T Assay

After size-exclusion chromatography, IAPP and the ApoE variants were mixed in 96-well microtiter plates (black walls and clear bottoms, Corning, New York, NY, USA, Cat. No. 3881) with final concentrations as indicated in the figures and with 40 µM Thioflavin-T (ThT) (Sigma-Aldrich, Saint Louis, MO, USA, cat # T3516) in PBS/EDTA buffer. The kinetics of ThT binding were recorded on a FLUOstar Omega microplate reader (BMG Labtech GmbH, Ortenberg, Germany) using an excitation wavelength of 440 nm and an emission wavelength of 480 nm, with 5 s shaking at 100 rpm before each cycle measurement at 15 min intervals. All experiments were performed at 37 °C. The experiments were repeated at least three times to verify the effect. Representative curves are shown by mean values of three technical replicates of the same experiment.

For the experiments probing the effect of ApoE at different stages of IAPP aggregation kinetics, the plate was prepared with 100 µL/well monomeric IAPP at 5 µM, including 40 µM ThT in PBS/EDTA buffer. A sample including ApoE from the beginning was denoted as time-0 (0 h). For the rest of the samples, the plate reader was paused at the indicated time points throughout the reaction. ApoE variants from concentrated stocks corresponding to 25 µM were added into the appropriate wells with minimum volume (2 µL/well) to a final concentration of 500 nM. Samples with no addition, as well as samples with the addition of the same volume of buffer, were used as controls.

### 2.3. Negative Staining and Transmission Emission Microscopy

Samples were sonicated for 45 s in a water bath before the negative staining. A total volume of 3.5 µL of samples was applied to 300 mesh formvar and carbon-coated, glow-discharged Ni grids and allowed to adhere for 5 min and then washed in MilliQ water to remove any excess sample. Grids were negatively stained three times for 10 s with 1.5% uranyl acetate (TAAB, Aldermaston, Berks, England). Samples were examined with a Talos L120 TEM microscope (120 kV) (FEI Thermo Fisher Scientific, Hillsboro, OR, USA) equipped with a Ceta CMOS 4k × 4k pixel camera (FEI Thermo Fisher Scientific) supported with the TEM Imaging & Analysis Software (TIA, FEI Thermo Fisher Scientific).

### 2.4. Cell Viability Assay

Human brain vascular pericytes were obtained from ScienCell (San Diego, CA, USA) and cultured according to the manufacturer’s recommendation. Briefly, the cells were grown on the poly-l-lysine-coated surface of culture flasks using pericyte medium (ScienCell #1201) supplemented with 2% FBS, 1% penicillin/streptomycin, and 1% Pericyte Growth Supplement (ScienCell #0503) in a 5% CO_2_ incubator at 37 °C. For viability experiments, the cells were plated in tissue culture-grade sterile 96 well plates (Eppendorf, Hamburg, Germany, #0030730135) at a density of 2500 cells/well in 100 µL complete culture medium and cultured for 24 h. Before treating the cells with IAPP and ApoE, the culture medium was changed to 50 µL serum-free medium and the cells were allowed to adapt for at least 1 h.

IAPP or ApoE monomers were isolated by size-exclusion chromatography in sterile PBS. The fractions were sterile filtered, and the concentrations were determined by NanoDrop (Thermo Scientific, Madison, WI, USA). Samples were diluted in serum-free culture medium and added to the cells in triplicate wells at the final concentration range 16 nM–1 µM. Cell viability was monitored and measured at 48 h, 72 h, and 144 h of incubation. Prior to measuring the viability at the given time intervals, the morphology of the cells was examined and documented with an EVOS FL cell imaging system (ThermoFisher Scientific, Waltham, MA, USA) using a phase-contrast filter. The cell viability was measured by WST-1 reagent (Roche Applied Sciences, Mannheim, Germany) according to the manufacturer’s instruction. Briefly, 10 µL/well of WST-1 was added and incubated at 37 °C for 2 h, and the absorbance of the WST-1 reduction product (formazan) was measured at 440 nm with 610 nm as the reference on a Tecan Infinite 200Pro plated reader (Tecan, Männedorf, Switzerland).

In a separate experiment, the viability of the cells was also assessed by treating the cells with pre-incubated samples of IAPP and ApoE. In this case, the samples were first incubated at 37 °C in culture medium without FBS. In a parallel plate of the same setup, we monitored the ThT binding of the samples under the same conditions to verify amyloid formation in the culture medium. The incubation was terminated when all the samples reached the plateau after 75 h (Figure S4). Afterward, the samples were transferred to the pericytes grown in 96-well assay plates and co-incubated for 48 h (Figure S5).

The data of 2 independent experiments, each with 3 technical replicates were analyzed by a graphing and statistical analyzing software GraphPad Prism 5.01 for Windows (GraphPad Software Inc, San Diego, CA, USA, and the viability of the cells was calculated as a percentage of the untreated control cells and presented as means ± SD. Statistical analysis was performed using 2-way ANOVA.

## 3. Results

### 3.1. ApoE Interferes with IAPP Aggregation Kinetics and Suppresses the Formation of Amyloid Structures

**Figure 1:**
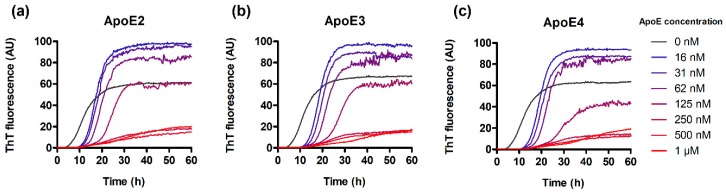
Thioflavin-T (ThT) binding kinetics of 5 µM islet amyloid polypeptide (IAPP) in the presence of the three apolipoprotein E (ApoE) variants: ApoE2 (**a**), ApoE3 (**b**), and ApoE4 (**c**). The ApoE variants were used at the concentrations indicated in the figure.

In order to assess the interference of ApoE with IAPP amyloidogenesis, we monitored the kinetics of IAPP aggregation in the presence of ApoE variants by using a ThT binding assay. IAPP aggregation kinetics displayed a sigmoidal curve shape, and under physiological conditions (i.e., pH 7.4 and 37 °C) the aggregation process of 5 µM IAPP alone showed a lag phase of approximately 5 h followed by about 15 h of elongation and finally reaching a steady state after 20 h of incubation (Figure 1).

The presence of ApoE led to a pronounced concentration-dependent prolongation of the lag phase. Interestingly, at steady-state, the samples containing lower concentrations of ApoE (16–62 nM) had significantly higher plateau levels (almost two-fold) compared to samples containing IAPP alone, whereas higher ApoE concentration (250 nM–1 µM) instead substantially suppressed the plateau compared to IAPP alone. However, no significant differences between the effects of the three ApoE variants were found (Figure 1a–c). Analysis of the results by Boltzmann sigmoid fitting also confirmed this observation (Figure S1a–c), where lag-phases, mid-points and plateau maximums show only marginal differences between the ApoE variants. Here, it is worth to mention that accurate calculation of lag phases is possible only at lower concentrations of ApoE (16–125 nM), while above these concentrations a parallel ThT-negative aggregation–reaction becomes significant resulting in both a change of the slope and a suppressed plateau at steady-state.

To verify that the effect induced by ApoE is specific and not the result of an unspecific interaction attributed to any protein added to the system, we also tested the effect of bovine serum albumin (BSA) on IAPP aggregation (Figure S2). The results show that BSA slightly interferes with IAPP aggregation but with a much weaker effect; importantly, BSA is also unable to suppress the plateau at a steady state (compare Figures S1 and S2).

### 3.2. ApoE Changes the Morphology of IAPP Fibrils

**Figure 2:**
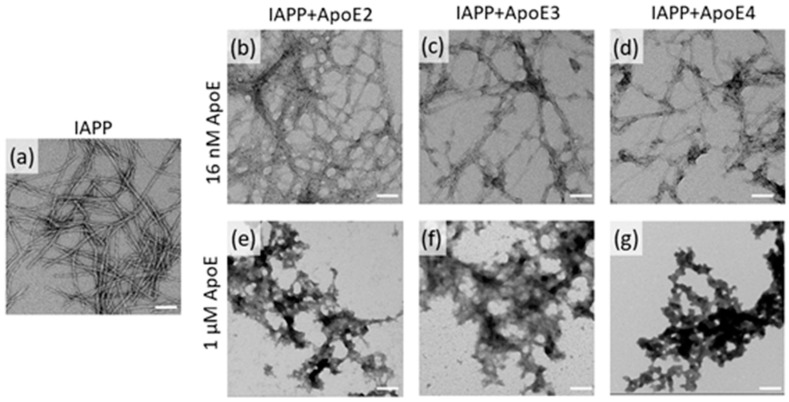
TEM images of IAPP samples incubated in the presence or absence of ApoE. Fibrils produced from 5 µM IAPP alone (**a**); from IAPP in the presence of 16 nM ApoE2, ApoE3, and ApoE4 (**b**,**c**,**d**); and from IAPP in the presence of 1 µM ApoE2, ApoE3, and ApoE4 (**e**,**f**,**g**). The scale bar is 100 nm. Larger fields of the images are shown in Figure S3.

In order to understand the nature of the structures influenced by ApoE, we analyzed the morphologies of endpoint IAPP species from ThT kinetics samples by using transmission electron microscopy (TEM). As shown in Figure 2a, IAPP alone formed thick, mature fibrils with a densely packed morphology of twisted filaments. In the presence of a low nanomolar concentration of ApoE (16 nM), the fibrils had a rather diffuse morphology with visible individual thin filaments (Figure 2b–d). However, formation of fibrillar structures was essentially abolished in samples containing a higher concentration of ApoE (1 µM), and only aggregates with a non-fibrillar morphology were observed (Figure 2e–g). Similar to the ThT kinetics results, no substantial differences were observed between the ApoE variants in induced morphological changes on IAPP.

### 3.3. ApoE Interferes with Nucleation and Elongation during IAPP Amyloid Formation

**Figure 3:**
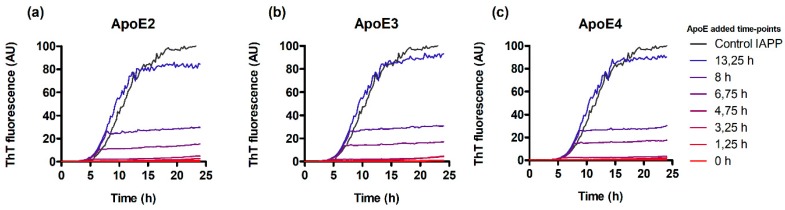
Effect of ApoEs on the elongation of IAPP during ThT binding kinetics. A total of 500 nM ApoE was added to the aggregating 5 µM IAPP at the indicated time points of the reaction. ApoE2 (**a**), ApoE3 (**b**), and ApoE4 (**c**).

In order to elucidate the mechanistic mode of interference of ApoE with the formation of IAPP amyloid, we probed different steps of the reaction by adding ApoE at different time points to an ongoing aggregation reaction. Here we used ThT assay again to monitor and control the process. As shown in Figure 3, the addition of ApoE during the lag phase (125–475 h) suppressed the reaction with similar efficiency as the presence of ApoE from the start (0 h) seen as a prolongation of the lag-phase.

Furthermore, the addition of ApoE to the exponential growth phase effectively stopped the reaction. Notably, the ThT signal intensity remained unaffected when adding ApoE from the beginning of the final plateau. All three variants of ApoE showed equal efficiency with no significant differences between the variants (Figure 3a–c).

### 3.4. ApoE Protects the Cells from IAPP-Induced Cytotoxicity

**Figure 4:**
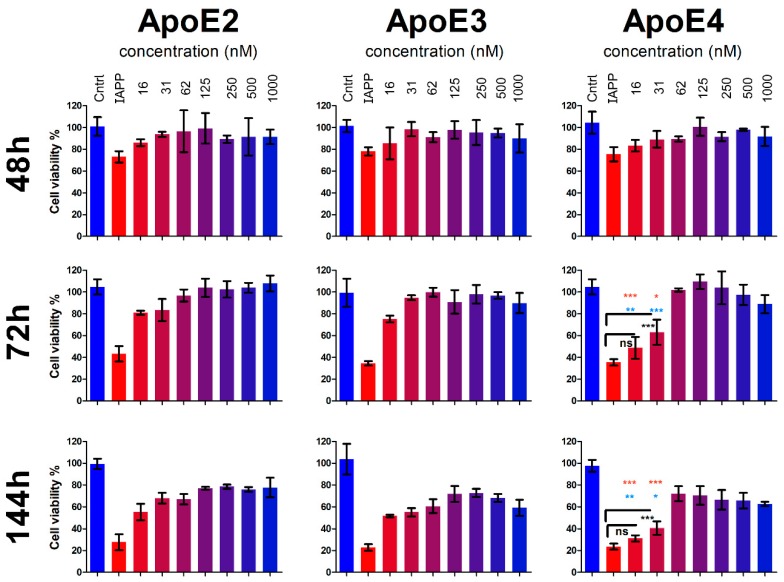
Influence of ApoEs on IAPP-induced cytotoxicity in human blood vessel pericytes. The viability of the cells treated with 10 µM IAPP alone or in combination with ApoE variants in the concentration range 1 µM–16 nM was analyzed after 48, 72, and 144 h of co-incubation with the cells. Control cells represent the samples treated with 1 µM ApoE only. The percentage of the viability is calculated based on untreated cells having 100% viability. The key statistical values are presented on the graphs. *p* < 0.05 (*); *p* < 0.01 (**); *p* < 0.001 (***). Red and blue colored asterisks represent comparison with ApoE2 and ApoE3, respectively.

To evaluate whether the inhibitory effect of ApoE on IAPP aggregation plays a protective or exacerbating role, we tested the influence of IAPP (10 µM) on cell viability in the absence or presence of all three ApoE variants at the concentration range of 16 nM–1 µM (Figure 4).

After 48 h of treatment, we observed a significant decrease in cell viability of the samples treated with 10 µM IAPP compared to untreated controls or to cells treated with ApoEs only (*p* < 0.01–0.001). The induced toxicity from IAPP was about 30% at 48 h, and this increased to about 65% at 72 h and to about 75% to 80% at 144 h (Figure 4). In essence, all the samples treated with ApoE along with IAPP showed higher cell viability than IAPP only-treated cells, with similar efficiencies for the ApoE variants observed at shorter incubation time (48 h).

However, upon prolonged incubation, a significantly weakened effect was observed with the lower concentrations of ApoE4 compared to the equivalent concentrations of ApoE3 and ApoE2. Particularly, at 16 nM and 31 nM ApoE4, the viability of the cells was only 48% and 63%, respectively, while in the presence of ApoE2 and ApoE3 the cell viability was in the range of 75% to 95% (*p* < 0.05 for ApoE4 vs. ApoE2 and *p* < 0.001 for ApoE4 vs. ApoE3). The differences between ApoE2 and ApoE3 at the given concentrations were not statistically significant.

During further incubation (up to 144 h), the overall protective effect from all ApoE variants was preserved, although with gradually decreased potential compared to 72 h. In contrast to the massive loss of cells treated with IAPP (only 20% to 25% viable cells remained), the percentages of the viable cells treated with ApoEs were mainly between 50% and 80%, with the overall highest viability in ApoE2-treated cells and the lowest for the cells treated with ApoE4, particularly at the lower concentrations. Here the lowest concentration was practically ineffective (only 31% viable cells, which was not significantly different from IAPP-treated cells in the absence of ApoE), and the 31 nM concentration was significantly less effective (40% viable cells) compared to the same concentration of ApoE2 (68% viability, *p* < 0.001) and ApoE3 (55% viability, *p* < 0.05) but still with significantly higher viability compared to IAPP only-treated cells (*p* < 0.01).

**Figure 5:**
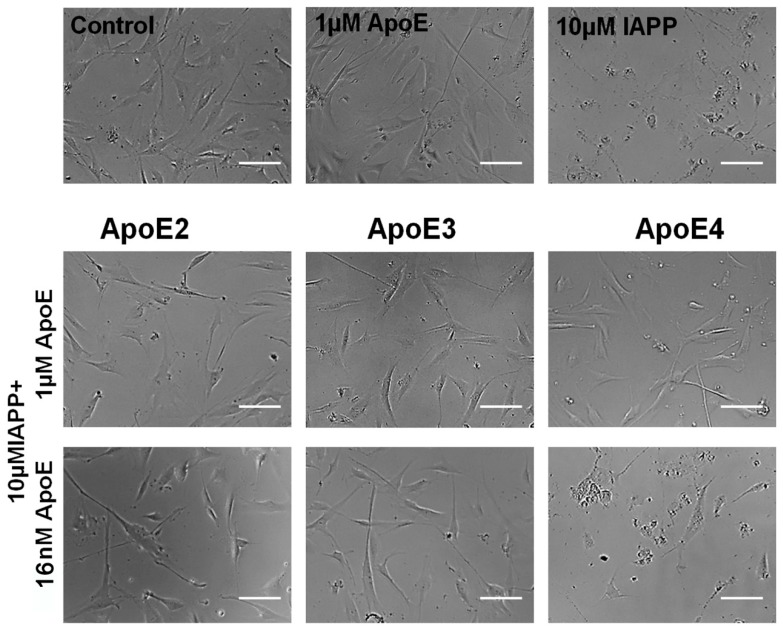
Morphology of the pericytes imaged under the phase-contrast microscope after 72 h treatment (scale bar is 100 µm). The upper panel represents controls (untreated cells) and cells treated with 1 µM ApoE only (here is the representative image from sample treated with ApoE4) and 10 µM IAPP only. Below are the images of the cells treated with 1 µM IAPP in combination with the indicated ApoE variants at the highest (1 µM) and the lowest (16 nM) concentrations used in the experiment.

The results of the cytotoxicity experiments were also confirmed by examination of the cells for morphological changes under the microscope. Figure 5 shows the representative images after 72 h treatment. The control untreated cells and the cells incubated with 1 µM ApoE alone showed similar, well-defined cellular morphology, while the cells treated with IAPP were largely degraded with loss of cellular borders and shape.

All ApoE variants at high concentrations (1 µM), as well as ApoE2 and ApoE3 at the lowest concentration, preserved healthy cellular morphology when added together with IAPP. Only the lowest concentration of ApoE4 did not rescue the cells from IAPP-induced toxicity, and the cells looked similarly degraded as those treated with IAPP only, which was in accordance with the results of the WST-1 assay (Figure 4).

## 4. Discussion

In the present work, we studied the ability of ApoE variants (ApoE2, ApoE3, and ApoE4) to interfere with IAPP amyloid formation in vitro and assessed their effect on IAPP-induced cytotoxicity on human blood vessel pericytes. By using a ThT-binding assay, we showed that in vitro interactions of ApoE variants with IAPP led to a concentration-dependent inhibition of IAPP amyloid formation.

IAPP-amyloid formation kinetics probed with ThT are characterized by a classical sigmoidal curve with three major phases—a lag phase, an elongation phase, and finally a saturation phase or plateau [61,62]. During the initial lag-phase, primary nucleation occurs with the formation of transient oligomeric species, but the extent of fibril formation has not yet reached a level that can be detected. The lag-phase subsequently converts into a phase of exponential fibril growth where elongation strongly dominates in the reaction [63,64]. The exponential increase in fibril growth here suggests a feedback mechanism where new sites amenable for fibril elongation are formed. This might be mediated by fibril breakage but possibly also via the process of fibril-catalyzed secondary nucleation as previously shown to dominate during fibril formation of Aβ [64,65]. The exact mechanism of secondary nucleation within this step has not been fully elucidated regarding IAPP, and it may also involve both fibril breakage and secondary nucleation.

Using the ThT assay, it is possible to experimentally target different parts of the reaction, and through the addition of ApoE at different time points to an ongoing aggregation reaction, its effect can be elucidated.

We showed that including ApoE from the absolute beginning induces a prolongation of the lag-phase (see Figure 1 and Figure 3—0 h). The effect is pronounced also at highly sub-stoichiometric ratios of ApoE versus IAPP indicating that ApoE targets oligomeric nuclei in preference of the monomer. An avid binding of ApoE to Aβ oligomers but not to monomer has recently been described [66], and simply based on the stoichiometry, a similar mechanism can be anticipated also here. The level of the plateau at steady state was also found to be affected in an unexpected manner, and regarding all ApoE variants, a significant increase in the ThT response was observed in the presence of low ApoE concentrations. However, using TEM analysis, we showed that the morphology of the fibrils was also significantly affected by lower concentrations of ApoE. Particularly, they became much thinner and diffuse compared to IAPP-only control fibrils. We suggest that the higher plateau is mediated by fibrillar morphology rather than the quantity of the formed fibrils. While the densely packed assembly observed in the control might mask the ThT binding sites, within the more diffuse fibrillar structures these sites are more exposed. It is noteworthy to mention that no significant differences were observed between the three ApoE variants in terms of ThT binding kinetics or morphology.

At higher ApoE concentrations the plateau at steady state was found to be effectively suppressed. This indicates that the fibrillar structure is disturbed in favor of alternative assemblies and an alternative, non-fibrillar, morphology could be verified by TEM. The interaction between IAPP and ApoE has been investigated in a previous study from 2008 [52]. However, our findings are in stark contrast to their results where it is claimed that IAPP fibrillation is prevented by low concentrations of ApoE4 but promoted by higher concentrations of ApoE4. We could not observe any promotion of the aggregation at higher concentrations of ApoE. This could possibly be explained by the differences in the handling of the peptide samples, which is a delicate work, and the methods for analysis have significantly been improved over the last decade. To further probe the mechanistic details, ApoE was added at different steps of the ongoing reaction. Addition of ApoE during lag-phase keeps this regime strongly suppressed, which could be expected based on the results in Figure 1. However, the addition of ApoE during the exponential phase, where elongation is the dominating process, the reaction is efficiently stopped (Figure 3). This result strongly suggests that elongation is affected and hence that ApoE has the possibility to bind at or in the vicinity of the fibrillar ends or in essence where elongation occurs on the structure. The addition of ApoE at steady state did not significantly affect the outcome of the end process, and the final plateau remained unaffected.

Taken together, the results suggest that ApoE specifically targets early and intermediate IAPP assemblies and prevents further elongation/maturation, while already formed mature fibrils remain unaffected by the addition of ApoE. No significant differences were observed between the ApoE variants, which suggests that the ability of ApoE to interfere with amyloid formation in vitro is isoform independent.

The inhibition of amyloid formation can have two different outcomes in a biological system. Depending on the origin of the inhibition, it can lead either to the stabilization and accumulation of early soluble aggregates/oligomers, which in turn would possibly lead to potentiated cytotoxic effect of these structures, or the inhibition can lead to the neutralization or at least suppression of the formation of cytotoxic species, thus protecting the cells from their harmful influence. Therefore, we further evaluated the effect of ApoE variants on IAPP-induced cytotoxicity. By using human blood vessel pericytes, we showed that all three ApoE variants at higher concentrations had protective effects against IAPP-induced toxicity and preserved the cellular morphology comparable to control (untreated) or ApoE-only treated cells. However, ApoE4 at lower concentrations lost its protective property, and the cells underwent degradation similar to the cells exposed to IAPP alone. The mechanism by which ApoE protects pericytes from amyloid-induced toxicity remains to be investigated further, with the focus on clarifying the question of whether ApoE mediates IAPP transport and degradation intracellularly or if it maintains IAPP bound in solution, thus preventing its interaction with the cells. However, it is clear from our results that besides the isoform differences, the concentration of ApoE is important in protecting the cells from cytotoxicity because at lower concentrations, all ApoE variants demonstrated reduced efficiency over time, although to a different extent and with the most pronounced decline being seen for the ApoE4 variant. The propensity of ApoE4 to aggregate shown recently [6] could contribute to this decline by reducing even more the concentration of functionally active molecules. These data are also consistent with the findings of significantly reduced expression of the ApoE4 variant in vivo [36,37], which might be an explanation for its association with AD pathology. On the other hand, ApoE4 has been shown to exacerbate the cytotoxic effect of Aβ in cellular models such as SK-N-SH and murine N2a neuroblastoma cells in culture [67], and knock out of ApoE in an AD mice model eliminates the signs of the disease [25,31,68], which makes the role of ApoE in amyloid-associated pathological processes more complicated and puzzling. Further comprehensive studies are needed to assess the effect of ApoE variants on amyloid-induced cytotoxicity using different cell types simultaneously.

## 5. Conclusions

Taken together, our results showing the inhibitory effect of ApoEs on IAPP aggregation, together with the inhibitory effect of ApoE on Aβ shown previously [20,21,30], indicate the possible generic property of ApoE to inhibit amyloid formation regardless of the isotype. However, discrepancies in the protective or potentiating effects of ApoEs might be explained by the specificity of cell types, which needs further in-depth investigation. The protective effect of ApoEs against IAPP-induced cytotoxicity in pericytes shown here supports the work by Bruinsma et al. [18] showing the protection of pericytes by ApoE against Aβ-induced cytotoxicity, where ApoE4 also had the weakest protective potential. These findings might be important keys in understanding the role of ApoEs in the pathological mechanisms involving IAPP and Aβ amyloids, particularly in association with vascular disorders. Moreover, they fill the gap in understanding the nature of ApoEs by showing their generic properties as inhibitors of amyloid formation and their possible dual role depending on the location and the cell type involved in the amyloid-associated pathologies. Our data indicate that ApoE4 shows slightly weaker protective potential on pericytes, which might be conditioned by isoform-specific loss of function. In addition, we showed that regardless of the isoform, the concentration of ApoE plays a critical role both in interfering with IAPP amyloid formation and in protecting the cells from IAPP-induced toxicity. This might have therapeutic significance, and correcting and balancing aberrant low ApoE levels to physiological levels might in turn partially compensate for the loss of protective function, especially for patients with the ApoE4 genotype.

## Supplementary Materials

The following are available online at https://www.mdpi.com/2218-273X/10/1/134/s1. Figure S1: Analysis of ThT binding kinetics results in Figure 1 by Boltzmann sigmoid fitting. Figure S2: Interference of BSA with IAPP amyloid formation. Figure S3: TEM images showing larger field samples presented in Figure 2. Figure S4 ThT binding kinetics of IAPP aggregation in pericyte culture medium. Figure S5: WST-1 assay of pericyte viability after treatment with pre-incubated samples. Figure S6: LC-MS analysis showing the quality of IAPP (a) and ApoEs (b,c,d). Supplementary figure legends.
