# Supplementary material for: Apolipoprotein E Interferes with IAPP Aggregation and Protects Pericytes from IAPP-Induced Toxicity
Source: Biomolecules. 2020 Jan 14;10(1):134. doi: 10.3390/biom10010134 (PMC7022431; doi:10.3390/biom10010134)
Supplement: Supplementary file 1 [file biomolecules-10-00134-s001.pdf]

Supplementary figure legends

Figure S1

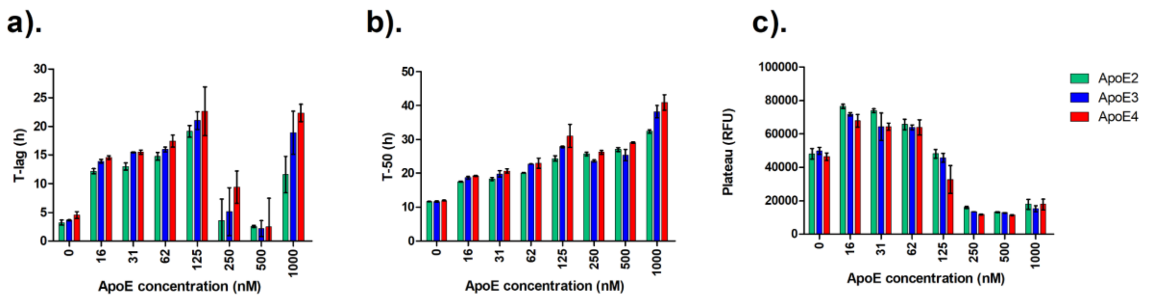

Suppl Figure1. Analysis of ThT binding kinetics results in Figure 1 by Boltzmann sigmoid fitting. (a) Time of the lag-phases (T-lag); (b) mid-points of the curves (T-50); and (c) plateau maximum. IAPP aggregation in the presence of ApoE2 (green), ApoE3 (blue) and apoE4 (blue).

Figure S2

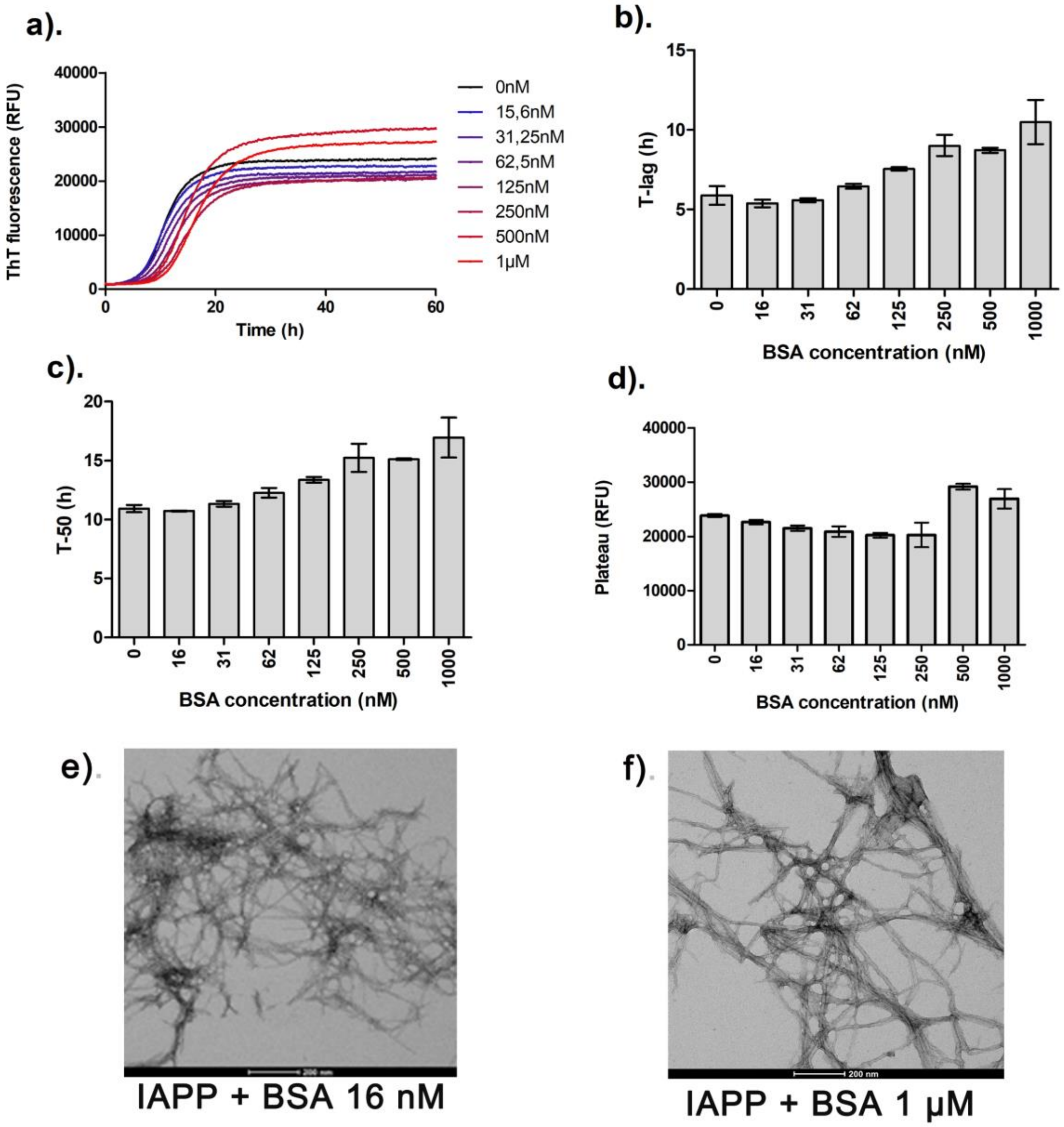

Suppl Figure2. Interference of BSA with IAPP amyloid formation. (a) ThT binding kinetics of 5 µM IAPP in the presence of BSA (16 nM-1µM); Boltzmann sigmoid analysis of **(b)** time lag-phases (T-lag); (c) mid-points of the curves (T-50); and (d) plateau maximum; TEM images of 5µM IAPP incubated in the presence of (e)16 nM BSA and (f) 1µM BSA.

Figure S3

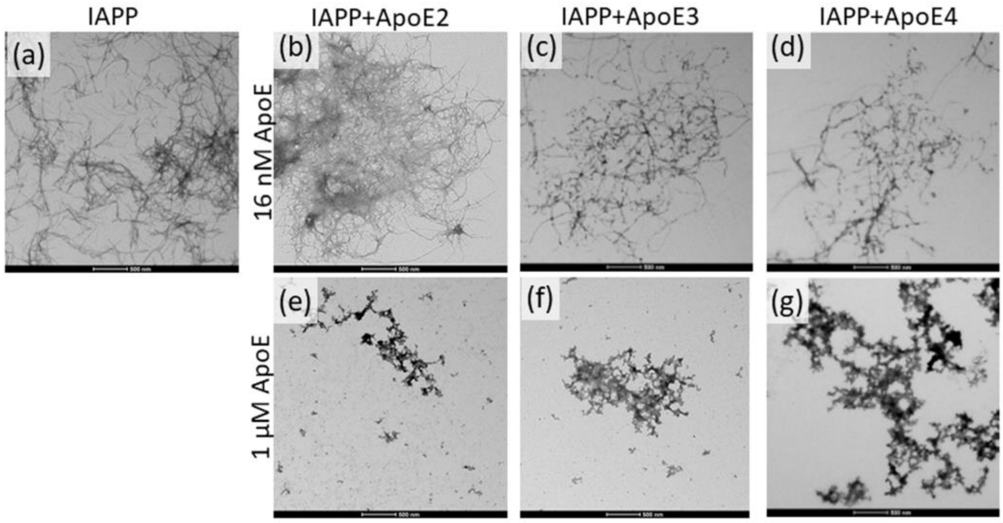

Suppl Figure 3 TEM images showing larger field samples presented in Figure 2. Fibrils produced from 5 µM IAPP alone (a), from IAPP in the presence of 16 nM ApoE2, ApoE3 and ApoE4 (b, c, d), and from IAPP in the presence of 1 µM ApoE2, ApoE3, and ApoE4 (e, f, g). Scale bar is 500 nm in all images.

Figure S4

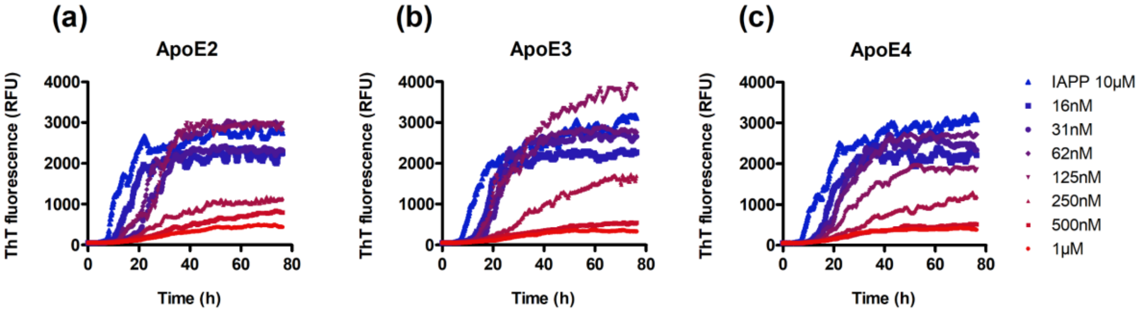

Suppl Figure 4. ThT binding kinetics of IAPP aggregation in pericyte culture medium. A total of 10 µM IAPP was incubated in the presence of the indicated concentrations ApoE2 (a), ApoE3 (b), and ApoE4 (c) in FBS-free pericyte culture medium at 37°C.

Figure S5

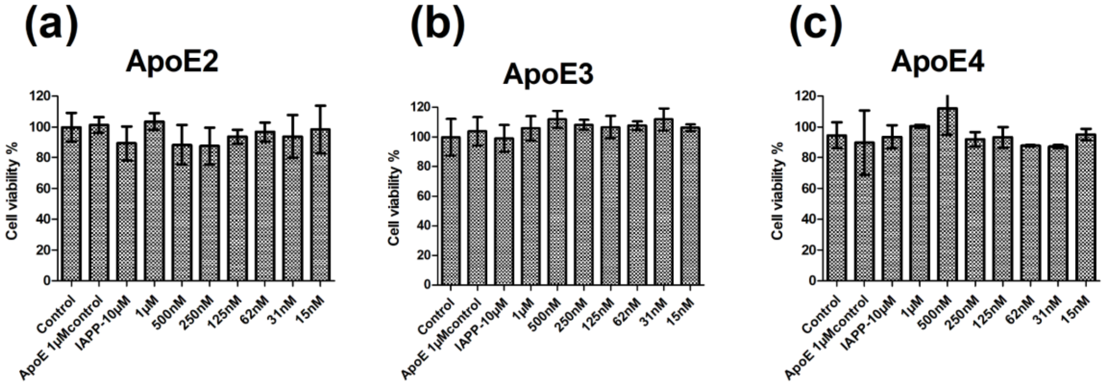

Suppl Figure 5. WST-1 assay of pericyte viability after treatment with pre-incubated samples. Cell viability was measured after 48 h of treatment with IAPP in the absence or presence of different concentrations of ApoE2 (a), ApoE3 (b), and ApoE4 (c) in FBS-free pericyte culture medium at 37°C.

Figure S6

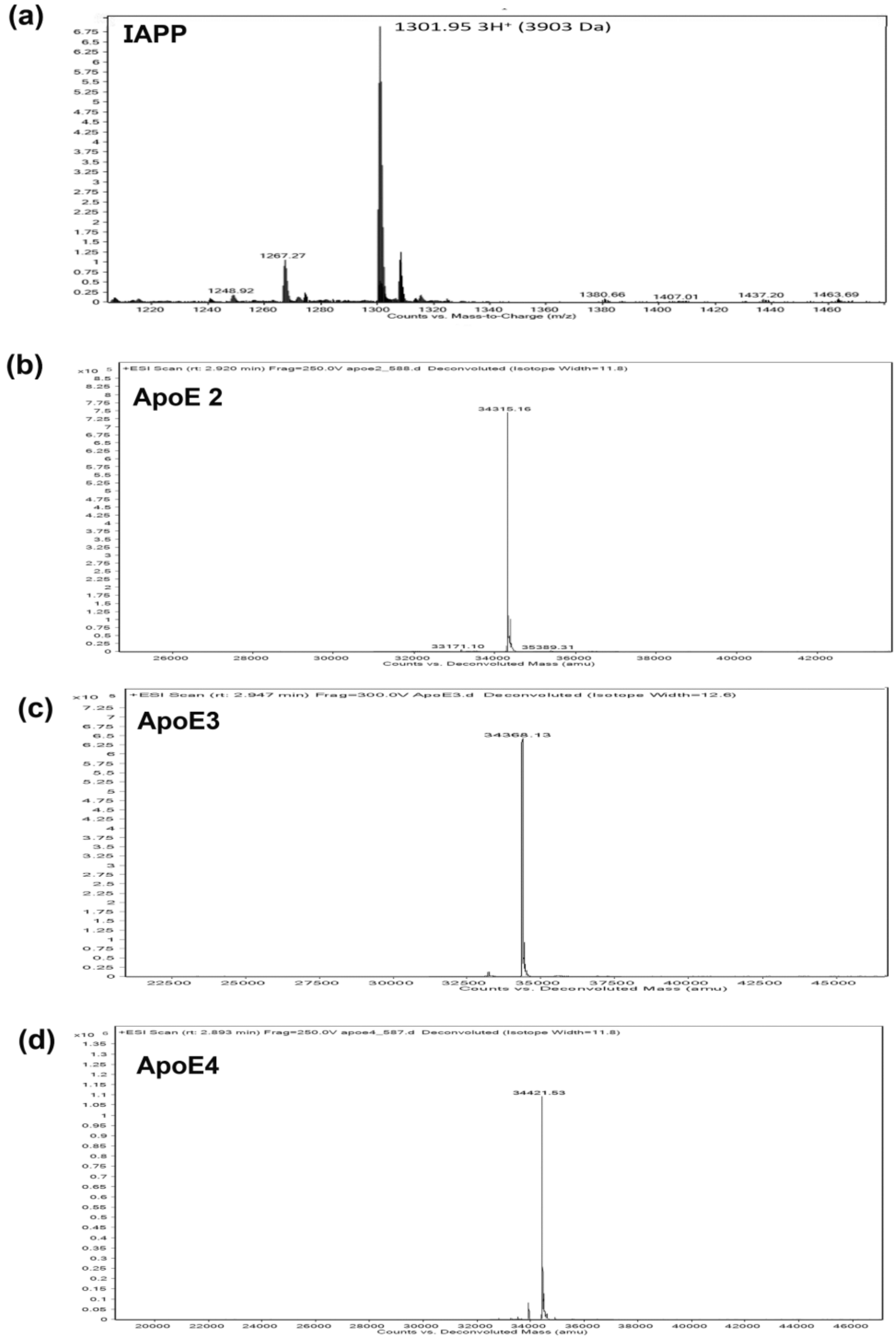

Suppl Figure 6. LC-MS analysis showing the quality of IAPP (a) and ApoEs (b, c, d).

The molecular weight and degree of labeling were verified by mass-spectroscopy (LC/MS TOF 6230B, Agilent, Santa Clara, CA, USA). All samples were dissolved in 5% acetic acid and desalted on an online C8 column (Agilent, Zorbax-C8, Kista, Sweden) using a water and ACN gradient containing 0.1% formic acid
